# Continuous Glucose Monitoring for Personalized Nutrition in Real-World Vively App Users: Retrospective Observational Study

**DOI:** 10.2196/80734

**Published:** 2026-04-29

**Authors:** Michelle R Jospe, Kelli M Richardson, Susan M Schembre

**Affiliations:** 1 Georgetown Lombardi Comprehensive Cancer Center Washington, DC United States

**Keywords:** precision health, digital health, metabolic health, personalized nutrition, blood glucose self-monitoring, biological feedback

## Abstract

**Background:**

The rising popularity of apps that sync with continuous glucose monitors (CGMs) reflects growing interest in on-demand, personalized care. These platforms combine real-time glucose biofeedback with self-monitored behaviors to optimize metabolic health among individuals with and without diabetes. However, little is known about user characteristics, engagement patterns, or factors associated with sustained use of CGM-integrated digital health apps in real-world settings.

**Objective:**

This study aimed to describe user demographics, CGM usage patterns, and food logging behaviors among Vively app users and to identify characteristics of sustained engagement with CGM wear and food tracking.

**Methods:**

We conducted a retrospective observational study of Vively app users between August 2021 and February 2025. Vively is a commercial digital health app that integrates with Abbott FreeStyle Libre CGMs to deliver personalized nutrition guidance. Users with at least 1 day of CGM wear were included. Primary outcomes were CGM wear duration (total days) and food logging engagement. Factors associated with engagement were identified using negative binomial regression for CGM wear and hurdle negative binomial models for food logging, adjusting for age, sex, BMI, baseline glucose, and device connectivity; the food logging model additionally adjusted for CGM wear category.

**Results:**

The analytical sample included 7647 users (4782/6905, 69.3% female, mean age 44.4, SD 10.9 years, mean BMI 27.8, SD 6.1 kg/m²). Users wore CGMs for a median of 15 (IQR 14-30) days, with 42.7% (3263/7647) completing one full wear period (13-15 days) and 30.3% (2315/7647) completing 2 or more wear periods (≥28 days). Most users (7013/7647, 91.7%) logged food at least once, with a median of 47 (IQR 18-91) food entries over 12 days. Food logging declined progressively during CGM wear (mean 63.2%, SD 8) and dropped sharply after sensor removal (mean 2.4%, SD 1.1). In multivariate models, higher baseline glucose was associated with longer CGM wear (incidence rate ratio [IRR] 1.15, 95% CI 1.13-1.17) but fewer food logging days (IRR 0.96, 95% CI 0.94-0.98). Connected device syncing showed the strongest association for both CGM wear (IRR 1.32, 95% CI 1.28-1.37) and food logging (IRR 1.45, 95% CI 1.39-1.51). Older age and female sex were associated with higher engagement in both behaviors.

**Conclusions:**

This large-scale analysis reveals distinct engagement patterns with CGM-integrated digital health applications. Food logging was largely concurrent with active CGM wear, dropping dramatically in CGM-free periods. The divergent associations of baseline glucose levels, with longer CGM wear but reduced food logging, may reflect different motivational drivers for passive monitoring versus active behavior tracking. These findings have important implications for designing sustainable digital health interventions that maintain user engagement beyond periods of biological feedback, though replication in more diverse samples and studies accounting for diabetes status and socioeconomic factors is needed.

## Introduction

In recent years, a growing number of companies have begun offering direct-to-consumer web- or app-based programs that are paired with continuous glucose monitors (CGMs) to optimize well-being and improve metabolic health. These programs represent an emerging application of precision nutrition: a technology-enabled approach that leverages individual variability in genetic, metabolic, phenotypic, and behavioral factors to optimize dietary recommendations and health outcomes [1,2]. By providing real-time, CGM-based biofeedback on glucose responses to meals and lifestyle behaviors, these platforms are increasingly marketed to individuals without diabetes as tools for metabolic optimization. Users are encouraged to adjust their diet, meal timing, and physical activity based on their personalized guidance to promote weight loss and glycemic stability, an indicator of metabolic health [3]. Early clinical trials have begun to demonstrate the effectiveness of combining CGM with personalized nutrition therapy on health outcomes among people without diabetes [4-6].

Advances in CGM technology and artificial intelligence, the precision health movement, and increasing device accessibility [5] have driven the expansion of commercial CGM-integrated apps. While CGMs were originally developed for insulin-dependent diabetes management, manufacturers have expanded access by releasing over-the-counter versions in several countries, enabling broader use among health-conscious consumers. Paired with mobile apps that translate sensor data into behavioral insights, CGMs are being positioned not only as medical devices but also as tools for health behavior change [6-9]. Yet most existing research on CGM-integrated apps has focused on diabetes management populations [10-12], with less attention to predominantly nondiabetic consumers using intermittent wear patterns for metabolic optimization. Despite the increasing interest in CGM-based mobile health apps, there is little empirical evidence describing who uses these tools, how they are used in everyday, nonclinical settings, and which factors are associated with sustained engagement. Most commercial platforms do not publicly report user characteristics or behavioral engagement metrics, leaving a critical gap in real-world evidence that limits both scientific understanding and the design of effective, scalable interventions.

This study addresses this gap by leveraging large-scale observational data from real-world users of the Vively app (Vively Health Pty Ltd), a commercial, CGM-integrated platform focused on personalized nutrition and lifestyle modification. We characterize user demographics, CGM usage patterns, and food and activity tracking, and identify associations with sustained engagement. In doing so, this work provides rare, real-world insight into how individuals interact with CGM-based feedback tools in consumer settings, with implications for the design and evaluation of precision digital health interventions.

## Methods

### Study Design

This was a retrospective observational study of Vively app users. The cohort entry window included users who initiated app use between August 2021 and November 2024. The follow-up window extended through February 2025, ensuring all users had at least 3 months of potential engagement data for analysis. Users were eligible for inclusion if they had at least one day of CGM sensor wear during the study period. The final analytical sample included 7647 users. This study followed STROBE (Strengthening the Reporting of Observational Studies in Epidemiology) guidelines for reporting observational research [13] (Multimedia Appendix 1).

### Vively CGM Program

**Figure 1:**
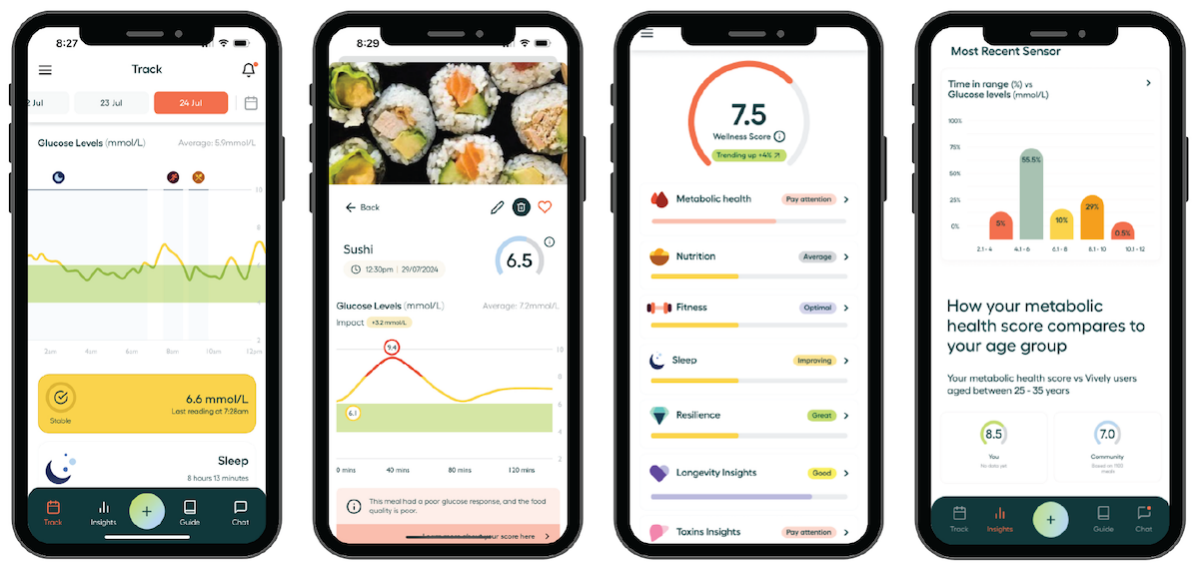
Screenshots of the Vively app.

The Vively app is a commercially available digital health application that integrates with the 14-day Abbott FreeStyle Libre 2 and 3 CGM. It delivers real-time glucose feedback, meal scoring, and behavioral guidance to support metabolic health, weight management, and disease prevention. Designed for users with and without diabetes, the app combines CGM data with self-reported food and activity logs, allowing users to observe how lifestyle factors affect glucose levels (Figure 1).

Vively includes meal-level scoring based on both nutrient quality and postprandial glucose responses. Users are encouraged to log meals during and between CGM wear periods to receive personalized feedback and track changes over time. The app can also sync with wearable devices to capture data on physical activity, sleep, and stress. Optional support from a registered dietitian is available as a paid add-on, offering one-on-one feedback for users seeking additional guidance.

Users typically begin their Vively experience by wearing a CGM sensor for 14 days to establish a baseline of glucose patterns and responses to food, activity, and lifestyle factors. During this period, users are encouraged to log meals and workouts to help the app generate personalized insights. After this initial wear period, users may continue to engage with the app without wearing a sensor. During these times, users can still log meals and receive guidance based on their previous CGM data and overall dietary patterns. Users are advised to repeat CGM wear approximately every 3 months as a refresher and to receive updated feedback. This intermittent approach is intended to balance the benefits of biological feedback with long-term usability and cost.

### Study Outcomes

User engagement was assessed using 2 outcomes: daily food logging and CGM wear duration. Daily food logging was treated as a continuous variable (total food logging days) and as a binary outcome indicating whether a user ever logged food. CGM wear duration reflects the total number of days with CGM data. In addition to modelling total CGM wear duration, we categorized wear duration to reflect the intended, intermittent, CGM use pattern (2 weeks, every 3 months): less than 1 full wear duration (<13 days), 1 full wear duration (13-15 days), 1 to 2 wear durations (16-27 days), and 2 or more wear durations (≥28 days). A CGM wear duration of 13-15 days will herein be referred to as 1 CGM wear, and a CGM wear duration of 15 or more days will be referred to as 2 (or more) CGM wears. Food logging served as our primary measure of app engagement, since users received feedback mainly on this behavior, while CGM sensor wear was used as a secondary measure of engagement.

Diet quality and diet glucose scores, as derived by the Vively app, were extracted as indicators of the average nutritional and glycemic quality of meals logged over the cumulative CGM wear and food logging duration. The diet quality score (range 0-10) reflects the nutritional composition of meals, including macronutrient distribution, degree of food processing, and whether the food contained alcohol. The diet glucose score (range 0-10) is derived from CGM data and captures postprandial glucose responses over a 2-hour window following each reported meal, and includes area under the curve, time-in-target range, and peak glucose levels. For each user, average scores were calculated across all eligible meals to summarize how meals aligned with the app’s metabolic goals. Higher scores for each diet-related outcome are more favorable.

Physical activity data were extracted from the Vively app as steps or workouts. Users with physical activity data were those who synced a connected device, such as a smartwatch or smartphone, to the Vively app. Step counts were inferred from any non-null step data linked to a third-party source (eg, Apple Health, Garmin). Workout data were collected from both third-party sources and user-logged events and were analyzed descriptively as a proxy for physical activity engagement.

### Statistical Analysis

We used descriptive statistics to summarize user characteristics, CGM wear, and app usage, reporting mean (SDs) or median (IQRs), as appropriate for the data distribution. Diet scores, step counts, and workout frequency were summarized descriptively and not included in modelling. Step counts were restricted to a plausible daily range of 500-100,000 steps. The lower bound was chosen to exclude days likely reflecting nonwear rather than genuine sedentary behavior, as very low counts are implausible for a full wear day [14]. Days with step counts more than 3 SDs below an individual’s mean were additionally removed; only below-mean outliers were excluded, as high step counts remain physiologically plausible.

CGM wear and food logging were the outcomes of interest, modeled separately as correlates of app engagement. Daily food logging rates were calculated across the first 14 days of the first CGM wear period and the first sensor-free period following it. The analytical sample on each day was restricted to users who had reached that wear day or break day. For wear days, users with shorter initial wear periods were excluded from subsequent days’ calculations. For break days, users who initiated a second sensor wear were excluded from subsequent days’ calculations, as their first sensor-free period had ended. To identify factors associated with food logging, we used a hurdle, negative binomial model. The hurdle model accounts for zero inflation and overdispersion in the count of logging days by modelling two components: (1) a logistic regression estimating the odds of ever logging food (≥1 day vs none), and (2) a zero-truncated negative binomial regression estimating the number of logging days among users who logged at least once. To reduce the influence of extreme values, food logging days were winsorized at the 99th percentile (111 days) prior to modelling, retaining all users in the analysis while reducing the influence of the 76 users (1%) with extreme logging durations [15]. Variables in both components included age, sex (reference: female), BMI, baseline mean glucose, and connected device use. The CGM wear category was included in the food logging model as an adjustment variable to account for the association between wear duration and food logging opportunity. As the CGM wear category represents a study exposure rather than a user characteristic, its coefficients are not presented in the regression forest plot.

Total CGM wear days were modeled using a standard negative binomial regression, using the same set of variables. Food logging days were excluded from this model to avoid adjusting for a potential downstream behavior. All continuous variables were modeled linearly and checked for collinearity. Exponentiated coefficients are reported as odds ratios or incidence rate ratios (IRRs), with corresponding 95% CIs. All regression models estimate associations between variables. As this is an observational study without randomization or experimental manipulation, findings should be interpreted as descriptive associations rather than causal effects.

User characteristics and engagement outcomes of interest were compared across the predefined categories of CGM use using pairwise tests. For continuous variables, pairwise independent 2-tailed *t* tests were conducted; for categorical variables, pairwise Fisher exact tests were used. All *P* values were adjusted for multiple comparisons using the Bonferroni correction. Effect size was calculated using Eta-squared (η^2^) for continuous variables and Cramér V for categorical variables. Users with missing values for any of the variables were excluded from regression models; no imputation was performed. Missing data were observed for age (n=274, 3.6%), BMI (n=293, 3.8%), and baseline mean glucose (n=218, 2.9%), resulting in 502 users (6.6%) excluded from both regression models, leaving an analytical sample of 7145. All analyses were conducted in R (version 4.2.2; R Foundation for Statistical Computing), using the *pscl* and *MASS* packages for count models.

### Ethical Considerations

The Georgetown University Institutional Review Board reviewed the study and determined this secondary analysis of deidentified data to be exempt (STUDY00008558). On account creation, Vively users agree to the Health Privacy Policy, which explicitly permits the use of deidentified operational data for research purposes. Users may withdraw consent at any time by opting out within the app or by contacting Vively support. Data extraction was performed by Vively prior to transfer in accordance with their privacy policy and the terms of the data-sharing agreement, which governed how opt-out requests were handled. Data were deidentified before transfer. Data were transferred via a secure file transfer protocol under a formal data-sharing agreement that specified permissible uses, storage requirements, and publication rights, and prohibited reidentification. No direct identifiers were available to investigators. No compensation was provided. Vively provided deidentified data exports under a data-sharing agreement and had no role in study design, analysis, interpretation, or the decision to submit. Investigators retained full control of the analytic code and results.

## Results

### Vively User Characteristics

The analysis included a total of 7647 individuals who used Vively with at least 1 day of CGM sensor wear (Table 1). The cohort was predominantly female, with roughly equal proportions in the normal (2555/7354, 34.7%), overweight (2518/7354, 34.2%), and obesity (2164/7354, 29.4%) BMI categories. Age ranged from 18 to 88 years. Most users were based in Australia (7091/7571, 93.7%), where Vively Health is headquartered.

**Table 1:** User, engagement, and behavioral characteristics across continuous glucose monitor (CGM) wear categories.

Characteristics		All	<1	1	1-2	≥2	*P* value		Effect size^a,b,c^	
Number of CGM wears, n (%)		7647 (100)	1298 (17.0)	3263 (42.7)	771 (10.1)	2315 (30.3)	—^d^		—	
CGM wear duration (days), mean (SD)		27.8 (37.1)	8.5^e^ (3.1)	14.6^f^ (0.7)	22.0^g^ (3.7)	59.1^h^ (55.6)	<.001		0.32^b^	
**Sex, n (%)**								.08		0.03^c^
	Male	2120 (30.7)	379 (33.3)	876 (29.2)	208 (31.2)	657 (31.4)	—		—	
	Female	4782 (69.3)	760 (66.7)	2126 (70.7)	459 (68.8)	1437 (68.6)	—		—	
Age (y), mean (SD)		44.4 (10.9)	41.2^e^ (10.4)	43.8^f^ (11.0)	44.2^f^ (10.7)	47.0^g^ (10.6)	<.001		0.03^b^	
BMI (kg/m²), mean (SD)		27.8 (6.1)	28.1^e^(5.8)	27.4^f^ (5.7)	28.1^e^ (6.1)	28.0^e^ (6.6)	<.001		0.00^b^	
Mean baseline CGM glucose (mmol/L)^i^, mean (SD)		5.8 (1.1)	5.7^e^ (1.0)	5.7^e^(0.8)	5.7^e^ (1.1)	6.0^f^ (1.4)	<.001		0.01^b^	
CGM days with food logging^i^, mean (SD)		55.7 (35.1)	50.7^e^ (36)	62.9^f^ (34.6)	50.4^e^ (33.5)	50.1^e^ (33.9)	<.001		0.03^b^	
Meals per day, mean (SD)		3.5 (1.4)	3.2^e^ (1.5)	3.7^f^ (1.5)	3.4^g^ (1.3)	3.4^g^ (1.3)	<.001		0.02^b^	
Vively diet quality score^j^, mean (SD)		8.1 (0.7)	8^e^ (0.9)	8.1^f^ (0.7)	8.1^f^ (0.7)	8.2^g^ (0.7)	<.001		0.01^b^	
Vively diet glucose score^k^, mean (SD)		8.7 (0.8)	8.8^e^ (0.7)	8.7^f^ (0.7)	8.8^e^ (0.7)	8.6^f^ (0.9)	<.001		0.01^b^	
Users with connected devices, n (%)		4668 (61)	685^e^ (52.8)	1886^f^ (57.8)	483^g^ (62.6)	1614^h^ (69.7)	<.001		0.13^c^	
Steps per day, mean (SD)		3831 (4024)	3769 (3999)	3803 (4084)	3954 (4216)	3854 (3910)	.90		0.00^b^	

^a^Effect size calculated using Eta-squared (η^2^) for continuous variables and Cramér V for categorical variables. These reflect different types of association and are not directly comparable.

^b^For η²: small ≈ 0.01, medium ≈ 0.06, large ≈ 0.14.

^c^For Cramér V (df=3): small ≈ 0.06, medium ≈ 0.17, large ≈ 0.29.

^d^Not applicable.

^e,f,g,h^Values with e, f, g, h superscript letters indicate statistically significant differences in pairwise comparisons between CGM wear categories (<1, 1, 1-2, ≥2 wears) based on 2-sided independent *t* tests or Fisher exact tests with Bonferroni correction.

^i^Calculated from hours 24 to 96 of continuous glucose monitor wear.

^j^A high diet quality score indicates better nutrient composition and less food processing.

^k^A high diet glucose score indicates a more stable and controlled glucose response, while a low score suggests potentially more rapid and significant spikes in blood sugar.

### CGM Wear Patterns

**Figure 2:**
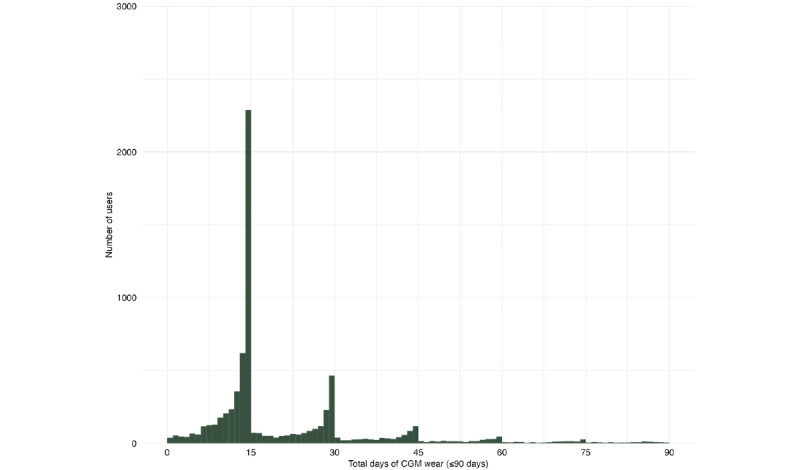
Distribution of total continuous glucose monitor wear days per user (N=7647). CGM: continuous glucose monitor.

**Figure 3:**
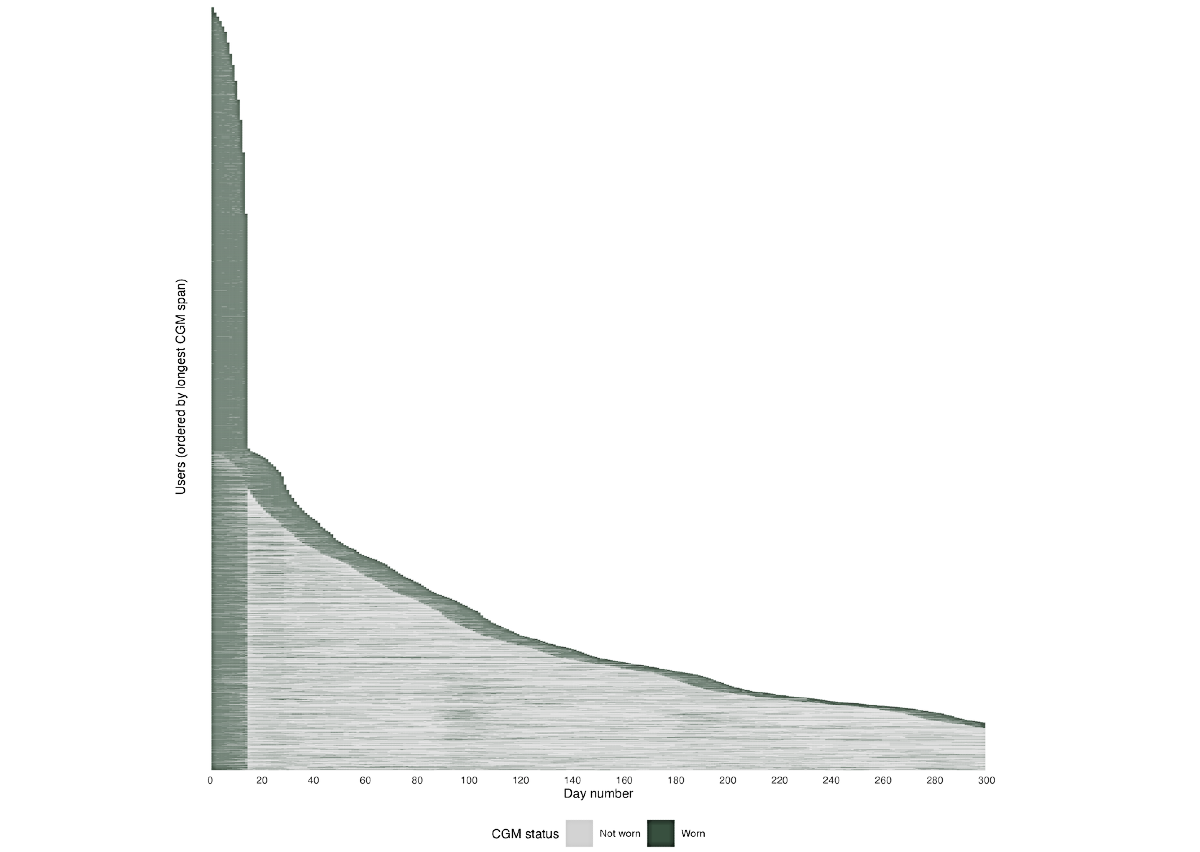
Continuous glucose monitor wear patterns among users over 300 days (N=7647). Each row represents an individual user, ordered by the longest span of CGM wear. Green tiles indicate days with continuous glucose monitor wear, and grey tiles indicate days without continuous glucose monitor wear. CGM: continuous glucose monitor.

Users wore a CGM for a median of 15 (IQR 14-30) days. There were 2 peaks in total wear durations: 42.7% (3263/7647) of users had 1 CGM wear (13-15 days), and 30.3% (2315/7647) of users had 2 or more CGM wears (≥28 days; Figure 2). For those with 2 CGM wears (either partial or full wears, 16-30 days; 1583/7647, 20.7%), the median number of days between CGM wears was 31 (IQR 6-85) days, and patterns varied widely (Figure 3). There were 141 users (1.8%) with 10 or more CGM wears (≥140 days). Reasons for partial CGM wear (<14 days; 2069/7647, 27.1%,) were not recorded.

### Food Logging

Most users (7013/7647, 91.7%) logged at least 1 food item. The median number of entries was 47 (IQR 18-91) items over a median of 12 (IQR 6-19) days. One hundred users logged on for at least 100 days, and 8 users logged food on more than 300 days. As with CGM wear, the most common cumulative food logging duration was 13-15 days (960/7647, 12.6%). On days with food entries, users reported an average of 3.5 (SD 1.4) meals per day.

Food logging declined progressively during CGM wear and dropped sharply after sensor removal. During the first 14 days of the initial CGM wear period, daily food logging rates averaged 63.2% (SD 8), declining from 72.3% (5519/7638) on the first wear day to 50.7% (2500/4934) on the 14th wear day. After sensor removal, daily food logging rates averaged 2.4% (SD 1.1), declining from 5.4% (415/7636) on the first day after sensor removal to 1.6% (95/6091) on the 14th day after sensor removal.

Diet scores were presented to users for each logged meal and were scored from 0 to 10, with higher scores suggesting better food choices. Diet quality scores, which were based on nutrient composition and degree of food processing, were available for 77.5% (421,418/543,476 meals) of logged meals across 6876 users. To receive a score, a meal needed to contain at least one ingredient with nutrient information. The cumulative, per-user mean diet quality score was 8.1 (SD 0.7). Diet glucose scores, based on postprandial glucose response, were available for 86.3% of logged meals (469,279/543,476 meals) across 6917 users. The cumulative, per-user mean diet glucose score was 8.7 (SD 0.8).

### Vively User, Engagement, and Behavioral Characteristics Across CGM Wear Categories

When comparing Vively users, engagement, and behavioral characteristics across CGM wear categories, most between-group differences were small in magnitude (η² ≤0.03; Cramér V ≤0.13) and should be interpreted cautiously given that statistical significance is expected across nearly all comparisons at this sample size (Table 1). Compared to users with 1 total CGM wear, users with 2 or more CGM wears were older (47.0 (SD 10.6) years vs 43.8 (SD 11) years, *P*<.05), they had the highest mean baseline CGM glucose, they reported fewer meals per day (on average), and had slightly higher Vively diet quality scores. Users with 1 total CGM wear also had the highest proportion of CGM days with food logging (62.9%, SD 34.6), with users in all other wear categories logging on a smaller proportion of their CGM days (50.1%-50.7%). Users with less than 1 CGM wear were the youngest users; they were least likely to have a connected device synced with Vively, they reported the fewest number of meals per day, and had the lowest Vively diet quality score. Users with 1-2 CGM wears were not markedly different from users with 1 CGM wear or 2 or more CGM wears.

### Steps and Workouts

Most users (4668/7647, 61%) connected Vively to a third-party device. The most common devices were the Apple Watch (3026/7647, 39.6%) and the Garmin watch (625/7647, 8.2%). Among those with connected devices, step counts were recorded on average for 80.6 (SD 79.2) days, with a mean daily step count of 3831 (SD 4024). Exercise logging was high, with 78.8% (6029/7647) users logging at least one workout. The number of workouts per user varied widely (median 28, IQR 7-93).

### Factors Associated With Food Logging and CGM Wears

Regression analyses examined associations between measured user characteristics and engagement, but could not control for unmeasured factors such as diabetes status, medications, or socioeconomic circumstances.

**Figure 4:**
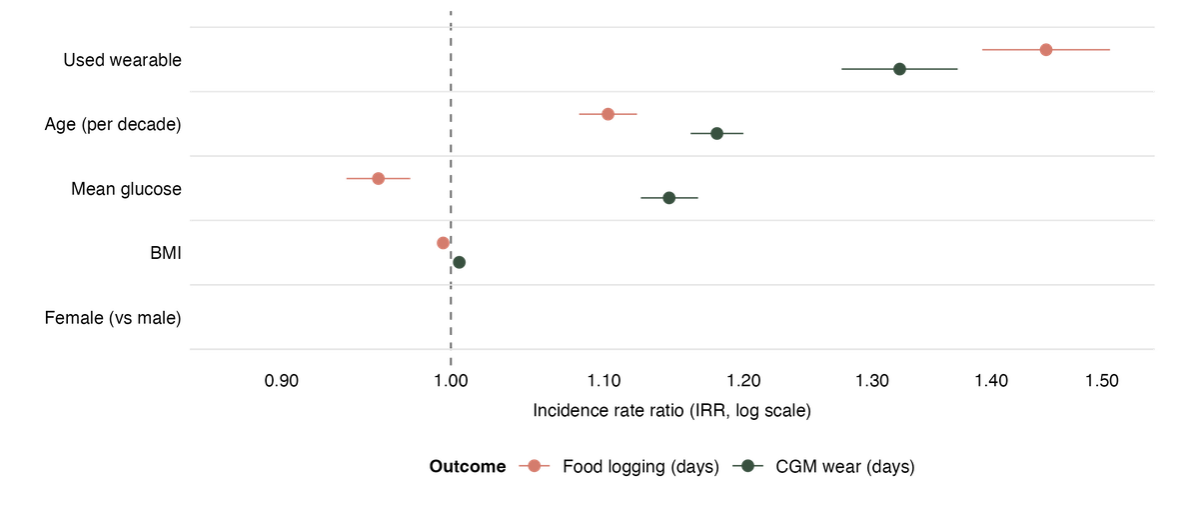
Factors associated with engagement behaviors. Incidence rate ratios with 95% CIs from negative binomial models of food logging days and continuous glucose monitor wear days. Incidence rate ratios are shown on a log scale; values >1 indicate positive associations. CGM: continuous glucose monitor; IRR: incidence rate ratio.

Figure 4 depicts the factors associated with app engagement, measured as food logging (total days) and CGM wear duration (total days). Baseline mean glucose showed divergent associations across outcomes, with higher glucose associated with greater CGM wear but reduced food logging. Device syncing, older age, and being female were associated with higher engagement for both outcomes. Associations with BMI were small and inconsistent across models.

Among the 7013 Vively users (91.7% of 7647) who logged food at least once, females logged 14% more days than males (IRR 1.14, 95% CI 1.09-1.19). Each additional decade of age was associated with an 10% increase in logging days (IRR 1.10, 95% CI 1.08-1.12), and each 1 mmol/L higher mean glucose was associated with 4% fewer logging days (IRR 0.96, 95% CI 0.94-0.98). Users with a connected device logged 45% more days than those without (IRR 1.45, 95% CI 1.39-1.51). Logging days were marginally lower with higher BMI (IRR 0.995, 95% CI 0.992-0.998).

CGM wear duration was positively associated with baseline glucose (IRR 1.15, 95% CI 1.13-1.17). Similar to food logging, use of a connected device was associated with more CGM days (IRR 1.32, 95% CI 1.28-1.37). CGM wear days were 8% higher among women than men (IRR 1.08, 95% CI 1.04-1.12) and were slightly higher among users with higher BMI (IRR 1.01, 95% CI 1.00-1.01) and older age (IRR 1.18 per decade, 95% CI 1.16-1.20).

## Discussion

### Principal Results

This large-scale observational analysis of Vively users offers a rare window into real-world use of a digital health app that integrates CGM with self-tracked behaviors. The cohort of 7647 users was predominantly female and middle-aged, with a broad range of BMIs and mostly normoglycemic glucose levels. Most users wore a CGM for 15 days, with distinct peaks in wear duration at 13 to 15 and 28 to 30 days, reflecting 1 or 2 sensor periods, respectively. Users with repeat CGM use (2 or more wears) tended to be older and had higher baseline glucose levels, though differences in user characteristics across CGM wear categories were generally small in magnitude. Food logging, used as an indicator of app engagement, was largely confined to periods of active CGM wear, declining progressively during the wear period and dropping sharply after sensor removal. Several user characteristics were associated with engagement, with some variation by outcome. Higher glucose levels were linked to longer CGM wear but fewer food logging days. Consistent associations of greater engagement across both behaviors included device syncing, older age, and identifying as female. BMI showed small and inconsistent associations with engagement.

The level of engagement among this cohort of Vively app users, specifically in food logging, was notable, even when accounting for potential self-selection bias. The observed level of self-directed adherence suggests a motivational factor, likely driven by the immediacy and personal relevance of glucose feedback. This aligns with prior research showing that combining food logging with self-monitoring of behavioral outcomes, such as self-weighing during weight loss efforts, is associated with higher engagement and greater goal attainment [16]. Other studies have found that personalized feedback independently improves adherence to food logging [17-19]. CGM-based feedback may provide particularly salient motivation, as it offers immediate, personalized insights into the physiological consequences of food choices. While our observational design cannot determine whether CGM feedback directly causes increased engagement or whether highly motivated individuals are more likely to both wear CGMs and log food consistently, previous research has shown that self-monitoring diet is itself a key behavior in achieving health goals and has been identified as one of the strongest predictors of weight loss [16,20-22]. Nevertheless, food logging dropped sharply after CGM removal, even though the Vively app continues to provide personalized dietary feedback in the absence of sensor wear, suggesting that engagement with active self-monitoring behaviors is closely tied to the availability of real-time biological feedback.

Several factors were consistently associated with overall engagement with the app, including both food logging and CGM wear. Device syncing emerged as one of the strongest relationships across both outcomes, suggesting that integration of multiple data sources may be associated with higher engagement by providing more complete and rewarding feedback. Previous research has found that integrating third-party devices, such as wearables, into interventions has been associated with improved engagement [23] and enhanced physical activity outcomes [24,25]. As wearable technology becomes more sophisticated and apps increasingly integrate multiple devices and data streams, the role of device connectivity in sustaining user engagement warrants further investigation. Understanding both how users interact with digital health technologies and whether that interaction supports behavior change is essential for designing effective interventions [26]. Older age was also associated with higher engagement in our sample, though this relationship is inconsistently observed across studies of nutrition app use [27]. Women were more likely to engage in both food logging and CGM wear. However, as is common in behavioral research, our sample included a greater proportion of women, which may have influenced these findings.

We found that baseline glucose showed divergent effects on engagement. While users with higher glucose were more likely to persist with CGM wear, they were paradoxically less likely to log food, potentially reflecting lower motivation for active diet tracking or greater burden of behavior change. Users with higher glucose levels may perceive greater value in the sensor data itself, focusing on monitoring rather than on modifiable behaviors such as dietary tracking. While these patterns suggest potential mechanisms, such as passive monitoring requiring less effort than active logging, or users with higher glucose prioritizing observation over intervention, our cross-sectional, observational design prevents us from determining the directionality or causality of these relationships. Longitudinal experimental studies would be needed to test these hypothesized mechanisms.

### Comparison With Prior Work

Our findings are consistent with emerging evidence on engagement patterns in CGM-integrated digital health applications. The divergent associations between baseline glucose and engagement behaviors, with higher glucose associated with greater CGM wear but reduced food logging, echo patterns observed in other studies. Kumbara et al [11] reported that diabetes management app users with baseline mean glucose >10 mmol/L (180 mg/dL) were substantially less likely to log food intake, exercise, sleep, and medication use. This suggests that elevated glucose may signal lower capacity or motivation for active self-monitoring despite greater clinical need. Dehghani Zahedani et al [10] reported similar engagement patterns, with nondiabetic participants showing better adherence to food and activity logging during a 10-day CGM intervention. While their superior metabolic outcomes (91.7% of nondiabetic participants improving time in range compared to 58.3% with type 2 diabetes) could reflect better engagement, metabolic differences and medication use likely contribute substantially to these outcome differences independent of engagement behavior. The consistent pattern of reduced engagement among those with elevated glucose nevertheless suggests that metabolic dysregulation may create barriers to effortful self-monitoring, whether through competing priorities, psychological burden, or lower self-efficacy.

Our results also support the expected finding that passive monitoring requires less sustained effort than active tracking. Böhm et al [12] documented this pattern in a diabetes management app, where users engaged more consistently with automated data entry, such as CGM, compared to manual input like food and medication logs. More recent real-world evidence confirms that CGM adherence patterns predict glycemic outcomes, with higher adherence associated with greater hemoglobin A_1c_ improvements [28]. We observed similar dynamics, with CGM wear showing more sustained engagement than food logging, particularly after the initial sensor period. While the lower effort required for passive monitoring compared to active tracking is self-evident, passive data collection methods also eliminate recall bias and reduce administrative burden compared to manual self-monitoring [29], which may explain their superior adherence patterns. Quantifying this difference in real-world commercial settings provides empirical support for prioritizing low-burden technologies in intervention design, particularly for individuals managing chronic conditions or elevated health risks who may have limited capacity for intensive self-monitoring.

Our study contributes to this evidence base by examining engagement in a predominantly nondiabetic, commercial app context. While prior research has focused primarily on clinical diabetes management platforms, we demonstrate that these engagement dynamics also occur in consumer-oriented precision nutrition applications targeting metabolic optimization. The progressive decline in food logging from an average of 63.2% during wear to 2.4% after sensor removal underscores how strongly engagement depends on the presence of immediate biological feedback. This pattern emerged even among self-selected, paying users presumably motivated to improve their health, which has implications for designing sustainable digital health interventions that must maintain user engagement beyond periods of wearable sensor use.

### Strengths and Limitations

As an observational study without a control group, randomization, or experimental manipulation, our findings represent associations rather than causal relationships. We cannot determine whether CGM feedback directly drives engagement behaviors or whether unmeasured factors influence both CGM adoption and self-monitoring behaviors. Additionally, we cannot establish temporal ordering, for example, whether baseline glucose influences subsequent engagement, or whether pre-existing engagement patterns predict glucose levels. To assess whether differential follow-up time could explain engagement differences across CGM wear categories, we compared available follow-up time across groups. Median follow-up time was broadly comparable across categories (45-70 weeks), and the correlation between follow-up duration and total wear days was negligible (Spearman ρ=0.06), suggesting that observed differences in wear reflect true engagement rather than observation time.

The dataset lacks information on diabetes status, medications, comorbidities, and socioeconomic status, factors that may influence both glucose levels and motivation to engage with health tracking technologies. These unmeasured factors could confound observed associations. As an observational dataset of paying users of a commercial wellness app, the sample is self-selected and skewed toward women and Australian residents, likely representing health-motivated early adopters of CGM technology rather than typical patients or the general population. While we characterize this as “real-world” data because it reflects naturalistic, voluntary app use in commercial settings (as opposed to controlled research conditions with prescribed protocols), findings may not generalize to nonpaying users, clinical populations, men, or other geographic regions. Furthermore, step count was based on data from users’ smartwatches or phones, without confirmation that devices were worn continuously, leading to potential underestimation when devices were removed or worn inconsistently.

However, the study’s strengths include its large sample size and ecological validity, providing rare insight into real-world use of a CGM-integrated digital health app outside of research or clinical settings. The availability of granular engagement data across both passive and active behaviors also enables a nuanced understanding of how users interact with biological feedback tools. While future studies with comprehensive health and socioeconomic data and more diverse samples are needed to enhance generalizability and disentangle these relationships, the current findings offer important baseline information about engagement patterns among early adopters of CGM-integrated wellness technology for hypothesis generation and intervention design.

### Conclusions

The rising popularity of apps that sync with CGMs reflects growing interest in on-demand, personalized care. This large-scale analysis of a commercial CGM-integrated app in naturalistic use underscores how user characteristics and engagement patterns among self-selected, paying users can inform the design of mobile-enabled tools that move healthcare beyond traditional models. While these findings reflect engagement among health-motivated early adopters, they provide important insights into the behavioral dynamics of CGM-integrated digital health interventions. As sensors become more affordable and longer-lasting [30], future research should explore not only how to sustain engagement beyond CGM use but also how long sensor wear continues to influence behavior. Identifying the behavioral drivers and specific features that support sustained engagement will be essential for developing interventions that are both scalable and effective [31-34]. This work should also expand to other apps and digital health platforms that use biofeedback to support personalized nutrition [35], to understand which tools and contexts most effectively promote lasting dietary behavior change. While our findings are associational and cannot establish causation, they provide valuable descriptive evidence of real-world usage patterns that can inform hypothesis generation for future experimental research. Gaps in our data, particularly the absence of diabetes diagnoses, medication information, comorbidity data, and socioeconomic indicators, highlight the need for future studies to comprehensively assess these factors to better understand how health status and social determinants influence CGM engagement patterns. Translating lessons from early adopters to broader, more diverse populations will require attention to access, usability, and behavioral support [33,36], especially for those who may benefit most from metabolic feedback but are less intrinsically motivated to actively self-monitor. Frameworks for understanding technology implementation, adoption, and sustainability can help guide efforts to scale these interventions across diverse settings and populations [37,38].

## Appendix

Multimedia Appendix 1 STROBE checklist.

## Abbreviations

CGM: continuous glucose monitor
IRR: incidence rate ratio
STROBE: Strengthening the Reporting of Observational Studies in Epidemiology

## Data Availability

Aggregate summary statistics and analysis code are available from the corresponding author on reasonable request. The raw data are not publicly available due to commercialization restrictions.

## References

[1] Berciano S, Figueiredo J, Brisbois TD, Alford S, Koecher K, Eckhouse S, Ciati Roberto, Kussmann Martin, Ordovas Jose M, Stebbins Katie, Blumberg Jeffrey B (2022). Precision nutrition: Maintaining scientific integrity while realizing market potential. Front Nutr.

[2] Livingstone KM, Ramos-Lopez O, Pérusse L, Kato H, Ordovas JM, Martínez JA (2022). Precision nutrition: a review of current approaches and future endeavors. Trends in Food Science & Technology.

[3] Suh S, Kim JH (2015). Glycemic variability: how do we measure it and why is it important?. Diabetes Metab J.

[4] Basiri R, Cheskin LJ (2024). Personalized nutrition therapy without weight loss counseling produces weight loss in individuals with prediabetes who are overweight/obese: a randomized controlled trial. Nutrients.

[5] Dixon W, Kim S, Levonian D, Gusz D, Fouladgar-Mercer S, Skyler JS (2025). Novel glucose metric "Latest Spike Time" correlated with weight loss at six months in people with obesity using the signos system. Diabetes Technol Ther.

[6] Richardson KM, Jospe MR, Bohlen LC, Crawshaw J, Saleh AA, Schembre SM (2024). The efficacy of using continuous glucose monitoring as a behaviour change tool in populations with and without diabetes: a systematic review and meta-analysis of randomised controlled trials. Int J Behav Nutr Phys Act.

[7] Jospe MR, Richardson KM, Saleh AA, Bohlen LC, Crawshaw J, Liao Y, Konnyu Kristin, Schembre Susan M (2024). Leveraging continuous glucose monitoring as a catalyst for behaviour change: a scoping review. Int J Behav Nutr Phys Act.

[8] Jospe MR, Kendall M, Schembre SM, Roy M (2025). Real-world effectiveness of glucose-guided eating using the data-driven fasting app among adults interested in weight and glucose management: observational study. JMIR Form Res.

[9] Veluvali A, Dehghani Zahedani A, Hosseinian A, Aghaeepour N, McLaughlin T, Woodward M, DiTullio Alex, Hashemi Noosheen, Snyder Michael P (2025). Impact of digital health interventions on glycemic control and weight management. NPJ Digit Med.

[10] Dehghani Zahedani A, Shariat Torbaghan S, Rahili S, Karlin K, Scilley D, Thakkar R, Saberi Maziyar, Hashemi Noosheen, Perelman Dalia, Aghaeepour Nima, McLaughlin Tracey, Snyder Michael P (2021). Improvement in Glucose Regulation Using a Digital Tracker and Continuous Glucose Monitoring in Healthy Adults and Those with Type 2 Diabetes. Diabetes Ther.

[11] Kumbara AB, Iyer AK, Green CR, Jepson LH, Leone K, Layne JE, Shomali Mansur (2023). Impact of a Combined Continuous Glucose Monitoring-Digital Health Solution on Glucose Metrics and Self-Management Behavior for Adults With Type 2 Diabetes: Real-World, Observational Study. JMIR Diabetes.

[12] Böhm A-K, Jensen ML, Sørensen MR, Stargardt T (2020). Real-world evidence of user engagement with mobile health for diabetes management: longitudinal observational study. JMIR Mhealth Uhealth.

[13] von Elm E, Altman DG, Egger M, Pocock SJ, Gøtzsche Peter C, Vandenbroucke JP, STROBE Initiative (2007). Strengthening the Reporting of Observational Studies in Epidemiology (STROBE) statement: guidelines for reporting observational studies. BMJ.

[14] Tudor-Locke C, Craig CL, Aoyagi Y, Bell RC, Croteau KA, De Bourdeaudhuij I, Ewald Ben, Gardner Andrew W, Hatano Yoshiro, Lutes Lesley D, Matsudo Sandra M, Ramirez-Marrero Farah A, Rogers Laura Q, Rowe David A, Schmidt Michael D, Tully Mark A, Blair Steven N (2011). How many steps/day are enough? For older adults and special populations. Int J Behav Nutr Phys Act.

[15] Flatt RE, Thornton LM, Tregarthen J, Argue S, Bulik CM (2025). How engagement changes over time in a digital eating disorder app: observational study. JMIR Mhealth Uhealth.

[16] Patel ML, Hopkins CM, Brooks TL, Bennett GG (2019). Comparing self-monitoring strategies for weight loss in a smartphone app: randomized controlled trial. JMIR Mhealth Uhealth.

[17] Turk MW, Elci OU, Wang J, Sereika SM, Ewing LJ, Acharya SD, Glanz Karen, Burke Lora E (2013). Self-monitoring as a mediator of weight loss in the SMART randomized clinical trial. Int J Behav Med.

[18] Patel ML, Wakayama LN, Bennett GG (2021). Self-monitoring via digital health in weight loss interventions: a systematic review among adults with overweight or obesity. Obesity (Silver Spring).

[19] Rivera-Romero O, Gabarron E, Ropero J, Denecke K (2023). Designing personalised mHealth solutions: an overview. J Biomed Inform.

[20] Turner-McGrievy GM, Dunn CG, Wilcox S, Boutté Alycia K, Hutto B, Hoover A, Muth Eric (2019). Defining Adherence to Mobile Dietary Self-Monitoring and Assessing Tracking Over Time: Tracking at Least Two Eating Occasions per Day Is Best Marker of Adherence within Two Different Mobile Health Randomized Weight Loss Interventions. J Acad Nutr Diet.

[21] Burke LE, Wang J, Sevick MA (2011). Self-monitoring in weight loss: a systematic review of the literature. J Am Diet Assoc.

[22] Raber M, Liao Y, Rara A, Schembre SM, Krause KJ, Strong L, Daniel-MacDougall Carrie, Basen-Engquist Karen (2021). A systematic review of the use of dietary self-monitoring in behavioural weight loss interventions: delivery, intensity and effectiveness. Public Health Nutr.

[23] Rayward AT, Vandelanotte C, Van Itallie A, Duncan MJ (2021). The association between logging steps using a website, app, or fitbit and engaging with the 10,000 steps physical activity program: observational study. J Med Internet Res.

[24] Tang MSS, Moore K, McGavigan A, Clark RA, Ganesan AN (2020). Effectiveness of wearable trackers on physical activity in healthy adults: systematic review and meta-analysis of randomized controlled trials. JMIR Mhealth Uhealth.

[25] Ferguson T, Olds T, Curtis R, Blake H, Crozier AJ, Dankiw K, Dumuid Dorothea, Kasai Daiki, O'Connor Edward, Virgara Rosa, Maher Carol (2022). Effectiveness of wearable activity trackers to increase physical activity and improve health: a systematic review of systematic reviews and meta-analyses. Lancet Digit Health.

[26] Yardley L, Spring BJ, Riper H, Morrison LG, Crane DH, Curtis K, Merchant Gina C, Naughton Felix, Blandford Ann (2016). Understanding and Promoting Effective Engagement With Digital Behavior Change Interventions. Am J Prev Med.

[27] Jakob R, Harperink S, Rudolf AM, Fleisch E, Haug S, Mair JL, Salamanca-Sanabria Alicia, Kowatsch Tobias (2022). Factors Influencing Adherence to mHealth Apps for Prevention or Management of Noncommunicable Diseases: Systematic Review. J Med Internet Res.

[28] Nemlekar PM, Hannah KL, Green CR, Norman GJ (2024). Association between adherence, A1C improvement, and type of continuous glucose monitoring system in people with type 1 diabetes or type 2 diabetes treated with intensive insulin therapy. Diabetes Ther.

[29] Germini F, Noronha N, Borg Debono V, Abraham Philip B, Pete D, Navarro T, Keepanasseril Arun, Parpia Sameer, de Wit Kerstin, Iorio Alfonso (2022). Accuracy and Acceptability of Wrist-Wearable Activity-Tracking Devices: Systematic Review of the Literature. J Med Internet Res.

[30] Huang X, Yao C, Huang S, Zheng S, Liu Z, Liu J, Wang Ji, Chen Hui-Jiuan, Xie Xi (2024). Technological Advances of Wearable Device for Continuous Monitoring of Glucose. ACS Sens.

[31] Michie S, van Stralen MM, West R (2011). The behaviour change wheel: a new method for characterising and designing behaviour change interventions. Implement Sci.

[32] Michie S, Richardson M, Johnston M, Abraham C, Francis J, Hardeman W, Eccles Martin P, Cane James, Wood Caroline E (2013). The behavior change technique taxonomy (v1) of 93 hierarchically clustered techniques: building an international consensus for the reporting of behavior change interventions. Ann Behav Med.

[33] Perski O, Blandford A, West R, Michie S (2017). Conceptualising engagement with digital behaviour change interventions: a systematic review using principles from critical interpretive synthesis. Transl Behav Med.

[34] Craig P, Dieppe P, Macintyre S, Michie S, Nazareth I, Petticrew M, Medical Research Council Guidance (2008). Developing and evaluating complex interventions: the new Medical Research Council guidance. BMJ.

[35] Richardson KM, Jospe MR, Saleh AA, Clarke TN, Bedoya AR, Behrens N, Marano Kari, Cigan Lacey, Liao Yue, Scott Eric R, Guo Jessica S, Aguinaga April, Schembre Susan M (2023). Use of Biological Feedback as a Health Behavior Change Technique in Adults: Scoping Review. J Med Internet Res.

[36] (2019). Guideline: Recommendations on Digital Interventions for Health System Strengthening.

[37] Greenhalgh T, Wherton J, Papoutsi C, Lynch J, Hughes G, A'Court C, Hinder Susan, Fahy Nick, Procter Rob, Shaw Sara (2017). Beyond Adoption: A New Framework for Theorizing and Evaluating Nonadoption, Abandonment, and Challenges to the Scale-Up, Spread, and Sustainability of Health and Care Technologies. J Med Internet Res.

[38] Greenhalgh T, Maylor H, Shaw S, Wherton J, Papoutsi C, Betton V, Nelissen Natalie, Gremyr Andreas, Rushforth Alexander, Koshkouei Mona, Taylor John (2020). The NASSS-CAT Tools for Understanding, Guiding, Monitoring, and Researching Technology Implementation Projects in Health and Social Care: Protocol for an Evaluation Study in Real-World Settings. JMIR Res Protoc.

